# Discovery and characterization of a novel chromosomally encoded aminoglycoside *O*-nucleotidyltransferase gene, designated *ant(9)-Ie*, in a strain of *Providencia*

**DOI:** 10.3389/fcimb.2026.1772530

**Published:** 2026-06-02

**Authors:** Lulu Huang, Zhigang Yang, Xiaolan Wu, Ruxi Hu, Xiaofang Hu, Wenjie Chen, Xuanjiao Mao, Susu Chen, Zhengqiao Li, Jie Gong, Junwan Lu, Qiyu Bao, Chunhan Song, Teng Xu, Hongqin Zhang

**Affiliations:** 1 Department of Laboratory Sciences, Pingyang Hospital of Wenzhou Medical University, Wenzhou, China; 2 Institute of Translational Medicine, Baotou Central Hospital, Baotou, China; 3 Institute of Biomedical Informatics, Wenzhou Medical University, Wenzhou, China; 4 School of Laboratory Medicine and Life Sciences, Wenzhou Medical University, Wenzhou, China; 5 Medical Molecular Biology Laboratory, School of Medicine, Jinhua University of Vocational Technology, Jinhua, China

**Keywords:** aminoglycoside, *ant(9)-Ie*, kinetic parameter, *Providencia rettgeri*, resistance gene

## Abstract

**Background:**

The complex aminoglycoside resistance mechanisms observed within the genus *Providencia* present formidable clinical challenges to the effective management of the infectious diseases caused by these species. In-depth research into the mechanisms of bacterial resistance to antimicrobials must be conducted to strengthen the response capabilities of healthcare facilities for infection prevention and control.

**Methods:**

Bacteria from wastewater from an animal farm were isolated by plate streaking. The minimum inhibitory concentrations (MICs) of the antimicrobials were determined using the standard agar dilution method. The function of the novel resistance gene *ant(9)-Ie* was elucidated using molecular cloning technology. ANT(9)-Ie was heterologously expressed, and its kinetic parameters were determined. The biological characteristics of the genome were systematically analyzed through whole-genome sequencing and a comparative genomic analysis, and a phylogenetic analysis of the genetic background for resistance gene-related sequences was performed.

**Results:**

In *Providencia rettgeri* P17, a novel resistance gene, designated *ant(9)-Ie*, conferring resistance against spectinomycin was identified. A comparative analysis revealed that among all the functionally characterized resistance proteins, ANT(9)-Ie had the highest amino acid (aa) sequence similarity to ANT(9)-Id (58.67%). Compared with the control strain *E. coli* DH5α (DH5α), the recombinant strain harboring *ant(9)-Ie* (pMD19-*ant(9)-Ie*/DH5α) showed a 64-fold increase in the MIC of spectinomycin. The results of the kinetic analysis of ANT(9)-Ie were generally consistent with the MIC for the cloned *ant(9)-Ie*, with a high spectinomycin catalytic efficiency [*k*_cat_/*K*_m_, (8.22 ± 1.24) × 10^2^ M^−1^·s^−1^). No new features were observed in the domains or motifs of the protein. The *ant(9)-Ie* homologous genes were found only in *Providencia* strains and exhibited a relatively conserved genetic environment. The *Providencia* strains whose genomes were available in the NCBI database were isolated from different sources, most of which were from human clinical specimens.

**Conclusions:**

In this study, a novel spectinomycin resistance gene, *ant(9)-Ie*, was identified, and its biological features were characterized. The identification of this novel resistance gene from *Providencia* might aid in the effective clinical treatment of infections caused by bacteria carrying this resistance gene in the future and paves the way for further research into the complexity of resistance mechanisms within microbial populations.

## Introduction

As members of the *Morganellaceae* family, bacteria of the genus *Providencia* are characterized by their ability to produce the enzyme urease (Liu et al., 2023). The first bacterium of the genus *Providencia* was isolated in 1904 (O’Hara et al., 2000). Currently, a total of 24 *Providencia* species have been recorded in the LPSN database (https://lpsn.dsmz.de/search?word=Providencia). The epidemiological characteristics of *Providencia* include significant environmental adaptability and host specificity, with a broad distribution and diverse infection types. This bacterium is commonly associated with diarrhea, with higher detection rates observed among children residing in tropical and subtropical regions. A study conducted in suburban central Kenya on hospitalized children with diarrhea reported a *Providencia* detection rate of 3.2%, with infections peaking significantly during the dry seasons (Dec.–Feb. and June–Sep.) of the year (59.3%) (Shah et al., 2016). Another study from a hospital in India reported that *Providencia* species accounted for 6.0% of the Gram-negative bacteria detected in urine samples, making it the fourth most common urinary tract pathogen (Sharma et al., 2022). Furthermore, the increasing trend of antibiotic resistance in *Providencia* has been concerning. An epidemiological and genomic study across six Romanian hospitals revealed 74 P*. stuartii* isolates carrying *bla*_NDM-1_ between 2021 and 2023. These isolates formed four interhospital clonal clusters, with ST46 as the predominant type, which has since spread to nine countries (Linkevicius et al., 2024). A study in Brazil revealed that *Providencia* carried multiple resistance genes and was transmitted among food-producing animals, suggesting that its resistance may extend beyond hospital settings and pose a potential threat to public health (Valiatti et al., 2024).

Aminoglycosides, as highly effective broad-spectrum antibiotics, offer numerous advantageous attributes and exhibit outstanding efficacy in treating life-threatening infections. This class of antibacterial agents displays potent inhibitory activity against aerobic, Gram-negative bacteria. The mechanism of action of aminoglycosides involves targeted binding to 16S rRNA within prokaryotic cells, which in turn halts bacterial protein synthesis, ultimately resulting in bactericidal effects (Aradi et al., 2020). Aminoglycoside resistance mechanisms generally include (a) aminoglycoside inactivation through *N*-acetylation, *O*-adenylylation or *O*-phosphorylation; (b) reduced intracellular concentrations of aminoglycosides due to alterations in outer membrane permeability (Nikaido, 2003), reduced transport across the inner membrane (Shakil et al., 2008), active efflux (Magnet et al., 2001), or drug sequestration (Magnet et al., 2003); (c) modification of the 30S ribosomal subunit target via mutagenesis (Musser, 1995); and (d) methylation of aminoglycoside binding sites (Magnet and Blanchard, 2005). Enzymatic modification is among the primary pathways leading to drug inactivation, in which aminoglycoside-modifying enzymes (AMEs) are classified into three major categories: aminoglycoside *N*-acetyltransferases (AACs), aminoglycoside *O*-nucleotidyltransferases (ANTs), and aminoglycoside *O*-phosphotransferases (APHs). ANTs are the smallest family of AMEs and can be divided into five major categories, namely, ANT(2’’), ANT(3’’), ANT(4’), ANT(6) and ANT(9), and transfer an adenyl group from ATP to a hydroxyl group of the antibiotic (Labby and Garneau-Tsodikova, 2013). An inducible AmpC β-lactamase can be produced in nearly all *Providencia* strains, while many isolates also demonstrate the potential for ESBL production in healthcare-associated environments (Malviya et al., 2024). This leads to the development of resistance to almost all β-lactam antibiotics (except carbapenems) in *Providencia*, making treatment extremely challenging. Consequently, treatment with alternative therapies, particularly aminoglycosides, is strongly recommended (Harris and Ferguson, 2012).

In this work, the resistance mechanisms of bacteria from different sources was explored through whole-genome sequencing and molecular cloning, and a novel aminoglycoside *O*-nucleotidyltransferase gene was identified from an environmental bacterium. The structure of the resistance gene-related sequences and the enzymatic properties of the resistance gene-encoded protein were further characterized.

## Materials and methods

### Bacteria and plasmids

*Providencia rettgeri* P17 was isolated from a wastewater pool at an animal farm in Wenzhou, Zhejiang Province, China. The strain was initially identified using the bioMérieux VITEK 2 Compact system (bioMérieux, Marcy L’Etoile, France). The species was further classified through a comparison of 16S rRNA gene homology and subsequently validated using average nucleotide identity (ANI) calculations (Konstantinidis and Tiedje, 2005; Richter and Rosselló-Móra, 2009) and in silico DNA-DNA hybridization (isDDH) (Auch et al., 2010). The strains and plasmids used in this study are listed in **Table 1**.

**Table 1:** Bacteria and plasmids used in this study.

Strain or plasmid	Relevant characteristic(s)	Source
Strain		
*Providencia rettgeri* P17 (P17)	Wild-type strain	This study
*E. coli* DH5α (DH5α)	Host for cloning the *ant(9)-Ie* gene	Our laboratory collection
*E. coli* BL21 (BL21)	Host for expressing ANT(9)-Ie	Our laboratory collection
*E. coli* ATCC 25922	Quality control strain for antimicrobial susceptibility testing	Our laboratory collection
pMD19-T-*ant(9)-Ie*/DH5α	DH5α carrying the recombinant plasmid pMD19-T-*ant(9)-Ie*	This study
pCold I-*ant(9)-Ie*/BL21	BL21 carrying the recombinant plasmid pCold I-*ant(9)-Ie*	This study
Plasmid		
pMD19-T	Cloning vector for the PCR products of the *ant(9)-Ie* gene with its upstream promoter region, ampicillin resistance	Our laboratory collection
pCold I	Expression vector for the PCR products of the ORF of the *ant(9)-Ie* gene, ampicillin Resistance	Our laboratory collection

### Antibiotic susceptibility testing

The minimum inhibitory concentrations (MICs) of the antimicrobial agents against all five strains [*P. rettgeri* P17, pMD19-*ant(9)-Ie*/DH5α, pMD19-T/DH5α, DH5α, and ATCC 25922] were determined using the agar dilution method on plates containing Mueller–Hinton (M–H) medium in accordance with the Clinical and Laboratory Standards Institute (CLSI, 2025) guidelines. *E. coli* ATCC 25922 was used as a quality control strain. The antimicrobial agents tested included spectinomycin, streptomycin, sisomicin, ribostamycin, tobramycin, gentamicin, kanamycin, neomycin, paromomycin and amikacin. The inhibitory concentrations (ICs) of spectinomycin against the four strains [pMD19-*ant(9)-Ie*/DH5α, pMD19-T/DH5a, DH5α, and ATCC 25922] were determined using the broth dilution method based on the procedures outlined in the CLSI M07 standard (CLSI, 2024) and ISO standard 20776-1:2019 (ISO, 2019). A single colony was inoculated into 2 mL of Mueller–Hinton (MH) broth and incubated at 37 °C with shaking for 18–24 hours. The bacterial suspension was then adjusted with fresh MH broth to an OD_600_ value of 0.125 (equivalent to a 0.5 McFarland standard) and subsequently diluted 1000-fold to prepare the final inoculum. A total of 100 μL of each serial dilution of spectinomycin (128, 192, 256, 384, 512, 768, 1,024, 1,536, 2,048 and 3,072 μg/mL) was added to each well of a 96-well plate, while 100 μL of MH broth was added to the blank control wells. Afterward, 100 μL of the prepared bacterial suspension was added to each well, and the plate was incubated at 37 °C for 14–16 hours. The background absorbance was corrected using the blank control wells, and the OD_600_ value of each well was measured using a microplate reader. The growth inhibition curve was generated by plotting the spectinomycin concentration (x-axis) against the OD_600_ value (y-axis) and fitting it using the four-parameter Hill equation (Yarlagadda et al., 2020). The corresponding equation and R^2^ value were added to the trend line for visualization. The IC_50_ and IC_90_ values were then calculated from the fitted curve. All the antibiotic susceptibility tests were performed in triplicate.

### Genome sequencing, assembly, annotation, and bioinformatics analysis

The genomic DNA of the isolate was extracted using the AxyPrep Bacterial Genomic DNA Miniprep Kit (Axygen Biosciences, Union City, CA, United States), and the genome was sequenced on next-generation sequencing platforms (PacBio RS II and Illumina HiSeq 2500) at Shanghai Personal Biotechnology Co., Ltd. (Shanghai, China). Sequencing data assembly and coding sequence (CDS) and resistance gene annotation were conducted using the methods described in a previous study (Yu et al., 2024). The average nucleotide identity (ANI) was calculated with fastANI v1.33 (Jain et al., 2018) and isDDH was conducted using the TYGS website (https://tygs.dsmz.de/). Clinker v0.0.24 (Gilchrist and Chooi, 2021) and Proksee (https://proksee.ca/) were used to analyze the genetic environment of *ant(9)-Ie* and its homologous genes. Multiple sequence alignment diagrams and phylogenetic trees were constructed using MAFFT v7.490 and MEGAX (Katoh and Standley, 2013; Kumar et al., 2018), respectively. JavaScript was used to estimate the molecular weight and the isoelectric point (pI) of ANT(9)-Ie (Stothard, 2000). The three-dimensional structure of the protein was first modeled using ColabFold (v1.6.1) (Mirdita et al., 2022) based on the AlphaFold2 algorithm (Jumper et al., 2021), from which the secondary structure features were subsequently extracted and analyzed. MGLTools (Morris et al., 2009) and AutoDock Vina (Eberhardt et al., 2021) were used for the molecular docking analysis, and the results were visualized using PyMOL (Janson and Paiardini, 2021).

### Cloning of the *ant(9)-Ie* gene

The *ant(9)-Ie* gene with its upstream promoter region was amplified by PCR using the primer sequences listed in **Supplementary Table 1**. The PCR product was inserted into the pMD19-T vector using a T4 DNA ligase cloning kit (Takara Bio, Inc., Dalian, China). The recombinant plasmid pMD19-T-*ant(9)-Ie* was introduced into competent DH5α cells via calcium chloride transformation. The transformant was selected using a Luria–Bertani agar plate supplemented with 100 μg/mL ampicillin. Further confirmation of the cloned sequence was performed using PCR and sequencing of the PCR products.

### Expression and purification of ANT(9)-Ie

The open reading frame (ORF) of the *ant(9)-Ie* gene was obtained by PCR using the primers listed in **Supplementary Table 1**, with the *Bam*HI and *Hind*III restriction sites incorporated into the forward and reverse primers, respectively, to obtain the ANT(9)-Ie protein. The PCR products and the expression vector pCold I were digested with both *Bam*HI and *Hind*III, and then the PCR products were ligated into the expression vector pCold I. The recombinant plasmid (pCold I-*ant(9)-Ie*) was transformed into *E. coli* BL21 cells. The transformants were selected on LB agar plates supplemented with 100 μg/mL ampicillin, and the inserts were subsequently verified by first-generation sequencing. The recombinant strain (pCold I-*ant(9)-Ie*/*E. coli* BL21) was cultured overnight at 37 °C in LB medium supplemented with 100 μg/mL ampicillin. The culture was subsequently transferred at a 1:100 dilution into fresh LB liquid medium and incubated until the OD_600_ ranged from 0.6–0.8. The induced expression and purification of the protein and analysis of the molecular mass and concentration of the protein were performed using the methods described in a previous publication (Yu et al., 2024).

### Kinetic studies of the enzyme ANT(9)-Ie

The enzymatic activity of ANT(9)-Ie was determined by coupling with glucose-6-phosphate dehydrogenase (G6PD), phosphoglucomutase (PGM), and UDP-glucose pyrophosphorylase (UGP). The reactions were monitored by measuring changes in the NADPH content at a wavelength of 340 nm with a SpectraMax Multi-Mode Microplate Reader (M5; Molecular Devices, USA). The final reaction system (200 μL) contained 2 mM ATP, 20 U/mL glucose-6-phosphate dehydrogenase, 20 U/mL phosphoglucomutase, 2 U/mL UDP-glucose pyrophosphorylase, 500 μM NADP, 500 μM glucose 1,6-bisphosphate, 500 μM UDP-glucose, 500 μM dithiothreitol (DTT), 10 mM MgCl_2_, 50 mM HEPES (pH 7.5), 75 nM purified ANT(9)-Ie and ddH_2_O. The reaction was initiated by adding 10 μL of varying concentrations of spectinomycin (7.5, 10, 12.5, 15, 20, and 25 μM, which were within the optimal substrate range based on the preliminary experiments) to the mixture, which had been preincubated for 5 minutes at 37 °C. The kinetic experiments were performed in triplicate. The kinetic parameters of the enzyme, *k*_cat_ and *K*_m_, were determined by fitting the data to the Michaelis–Menten equation using Prism software (v8.0.2) (Chen et al., 2019).

### GenBank accession numbers

The sequences of the chromosome and *ant(9)-Ie* of *Providencia rettgeri* P17 were deposited in the NCBI nucleotide database with accession numbers JBTYMD000000000.1 and PX485636.1, respectively.

## Results and discussion

### Identification of novel candidate resistance genes

In a recent study on the resistance profiles of environmental bacteria on animal farms in Wenzhou, China, approximately 200 bacteria were isolated from wastewater, sewage and soil samples. The MICs of various antimicrobial agents, including 10 aminoglycosides, against the bacteria were determined, and the whole genomes of these bacteria were sequenced. Notably, the annotated genes included several hypothetical aminoglycoside resistance genes, such as *aac(2’)-IIb-, aph(6)-Id-, aac(6’)-If-, aac(3)-IIIb-, aph(6)-Ic-, aadA5-, ant(9)-Ia-* and *aph(3’)-Ia-*homologous genes. The amino acid (aa) sequence similarity of the proteins they encoded with those proteins encoded by the functionally known aminoglycoside resistance genes was less than 80.0%. Several of the genes were randomly selected for molecular cloning and a systematic evaluation of their resistance phenotypes.

### Functional characteristics of the *ant(9)-Ie* gene

Each sequence containing the open reading frame (ORF) and promoter region was cloned and inserted into the pMD19-T vector to characterize the functions of the hypothetical resistance genes. The recombinant plasmid was then transferred into the recipient DH5α. As a result, an *ant(9)-Ic* homologous gene (named *ant(9)-Ie* in this study) conferring resistance to spectinomycin was successfully identified (**Supplementary Figure 1A**). The MIC of spectinomycin (2,048 mg/L) was high in DH5α harboring the recombinant plasmid (pMD19-*ant(9)-Ie*/DH5α), with an increase of 64-fold compared with that of the control strain (pMD19/DH5α, 32 mg/L), while the MICs of the other 9 aminoglycosides tested remained unchanged. The IC_90_ and IC_50_ of spectinomycin for pMD19-*ant(9)-Ie*/DH5α were 1,510 and 754.2 mg/L, respectively (**Table 2**). Compared with their recipients, recombinant strains carrying *ant(9)-Ib*, *ant(9)-Ic* and *ant(9)-Id* exhibit spectinomycin MICs that are increased 64-fold (LeBlanc et al., 1991; Kanchugal P and Selmer, 2020), 16-fold (Sheng et al., 2023), and 64-fold (Yu et al., 2024), respectively. It revealed that the increase in the MIC of spectinomycin was 4-fold greater for the recombinant strain carrying *ant(9)-Ie* than that observed in the recombinant harboring the *ant(9)-Ic* gene. The *ant(9)* family currently consists of five members, *ant(9)-Ia* to *ant(9)-Id* and *spd*, as determined from the CARD database. The resistance phenotype of *ant(9)-Ie* was the same as that of these *ant(9)* genes (Sheng et al., 2023; Wendlandt et al., 2013). These findings further confirmed that *ant(9)-Ie* might be a member of the *ant(9)-I* family.

**Table 2:** MICs of 10 aminoglycoside antimicrobials against 5 strains (mg/L).

Antimicrobial	P17	pMD19-*ant(9)-Ie*/DH5α			pMD19-T/DH5a			DH5α			ATCC 25922		
	MIC	MIC	IC_90_	IC_50_	MIC	IC_90_	IC_50_	MIC	IC_90_	IC_50_	MIC	IC_90_	IC_50_
Spectinomycin	≥ 2,048	2048	1,510	754.2	32	19.97	18.43	32	21.55	19.31	32	22.14	20.88
Streptomycin	256	2	–	–	2	–	–	2	–	–	4	–	–
Sisomicin	8	≤ 0.25	–	–	≤ 0.25	–	–	≤ 0.25	–	–	≤ 0.25	–	–
Ribostamycin	1,024	2	–	–	≤ 2	–	–	2	–	–	4	–	–
Tobramycin	8	0.25	–	–	0.5	–	–	0.25	–	–	0.5	–	–
Gentamicin	8	0.25	–	–	0.25	–	–	0.25	–	–	0.5	–	–
Kanamycin	512	1	–	–	1	–	–	1	–	–	2	–	–
Neomycin	32	1	–	–	1	–	–	1	–	–	1	–	–
Paromomycin	512	2	–	–	2	–	–	2	–	–	4	–	–
Amikacin	2	1	–	–	1	–	–	1	–	–	2	–	–

“-”, not determined; “MIC”, minimum inhibitory concentration; “IC_50_”, the concentration of inhibitor required to achieve approximately 50% inhibition; “IC_90_”, the concentration of inhibitor needed to achieve approximately 90% inhibition.

### The genome features and species classification of the P17 isolate

The novel resistance gene *ant(9)-Ie* was encoded by the isolate P17. The complete genome sequence of the P17 isolate was obtained to precisely analyze the molecular structure of the novel resistance gene-related sequence, and it consists of a single 4,646,596 bp chromosome (without a plasmid), with a GC content of 40.41% and 4,242 predicted ORFs (**Table 3**). The whole-genome sequencing and genome assembly quality metrics of this isolate are described in **Supplementary Table 2**. A total of 23 resistance genes, exhibiting ≥ 90.0% similarity to the functionally characterized resistance genes, were annotated from the genome. In addition to 3 copies of the disinfecting agent resistance gene *qacEΔ1*, the other 20 genes confer resistance to 8 classes of antimicrobials, including 6 conferring resistance to aminoglycosides (*aac(3)-IV*, *aph(4)-Ia*, *aac(6’)-Ib*, *aadA2*, *ant(3^”^)-IIa* and *aph(3’)-Ia*), 1 conferring resistance to cephalosporin (*bla*_CTX-M-55_), 1 conferring resistance to phenicol (*floR*), 1 conferring resistance to lincosamide (*lnuF*), 5 conferring resistance to sulfonamides (2 *sul2* and 3 *sul1*), 2 conferring resistance to diaminopyrimidine (*dfrA1* and *dfrA12*), 2 conferring resistance to macrolides (*msrE* and *mphE*), and 1 conferring resistance to rifamycin (*arr-3*).

**Table 3:** General features of the *P. rettgeri* P17 genome.

Description	Chromosome
Size (bp)	4,646,596
GC content (%)	40.41
Predicted coding sequences (CDSs)	4,242
Known proteins	2,753
Hypothetical proteins	1,489
Protein coding (%)	97.67
Average ORF length (bp)	923.8
Average protein length (aa residues)	311.2
tRNAs	78
rRNA operons	(16S-23S-5S) × 7, (16S-23S-5S-5S) × 1

“aa”, amino acids.

The species was classified by performing 16S ribosomal RNA gene homology, isDDH and ANI analyzes. The 16S ribosomal RNA gene homology analysis revealed that the sequence of the isolate P17 was most similar to that of *Providencia* sp. PROV040 (GenBank accession number CP120530.1, 100% identity and 100% coverage), followed by *Providencia rettgeri* (GenBank accession number CP039844.1, 100% identity and 99.93% coverage). For isDDH analysis, the isolate P17 genome shared an isDDH of 87.0% with that of *Providencia hangzhouensis* PR-310 (an informally species-classified bacterium, GenBank accession number CP135052.1), followed by *Providencia rettgeri* NCTC 11801 (GenBank accession number GCA_900455085.1) with an isDDH of 69.1% (the threshold is 70%). However, the genome sequence of the isolate P17 shared 98.65% ANI with the type strain *Providencia rettgeri* DSM1131^T^ (PRJNA29299), which is greater than the cutoff value of ≥ 95.0% for classifying a bacterial species. ANI has been established as the gold standard for the classification of bacterial species (Richter and Rosselló-Móra, 2009) and is used by the NCBI to evaluate prokaryotic genome classification systems (Ciufo et al., 2018). Therefore, based on the results described above, particularly the ANI results, the isolate P17 might be *Providencia rettgeri* and was thus designated *Providencia rettgeri* P17.

### Distribution of *ant(9)-Ie*-like genes and structural characterization of *ant(9)-Ie*-related sequences

*ant(9)-Ie* is 762 bp in size and encodes a 253-aa protein with a theoretical pI of 4.70 and a molecular weight of 28.58 kDa. The ANT(9)-Ie aa sequence was employed as a query to screen the NCBI nonredundant protein database for homologous sequences to investigate the distribution of *ant(9)-Ie-*like genes. A total of 45 homologs sharing ≥ 98.56% similarity with ANT(9)-Ie were available, with one predicted protein (CP120530.1) displaying the greatest (100%) identity to ANT(9)-Ie. The other predicted proteins shared only 76.20% or less similarity with ANT(9)-Ie. No protein with between 76.20% and 98.56% similarity was found. These 46 *ant(9)-Ie*(-like) genes (including the one identified in this study) were exclusively restricted to the genus *Providencia*, predominantly to *P. rettgeri* and unclassified *Providencia* strains, and were derived from diverse sources. Only one isolate (2.17%, 1/46) from this work was obtained from an environmental source, while the others were mainly obtained from human clinical (91.30%, 42/46) and animal (6.52%, 3/46) sources (**Supplementary Table 3**). This study demonstrated that *ant(9)-Ie*-like genes are present not only in environmental bacteria but also in those from animal and human sources, suggesting that these resistance determinants may be disseminated indirectly through environmental contamination, the food chain, or close human–animal contact. Such transmission pathways underscore the critical need for sustained surveillance of spectinomycin resistance. Although spectinomycin is relatively rarely used in human clinical settings, it remains a valuable agent in both veterinary and human medicine, particularly for managing certain livestock infections and selected clinical indications. These findings reinforce the importance of the One Health approach, which integrates antimicrobial resistance (AMR) monitoring across humans, animals, and the environment. Surveillance of farm wastewater isolates, in particular, can serve as an early warning system to detect resistance genes before they emerge in clinical pathogens. Accordingly, a comprehensive, One Health-based monitoring framework spanning the environmental, animal, and clinical domains is essential to preserve the clinical efficacy of spectinomycin and to curb the cross-sectoral spread of resistance.

**Figure 1:**
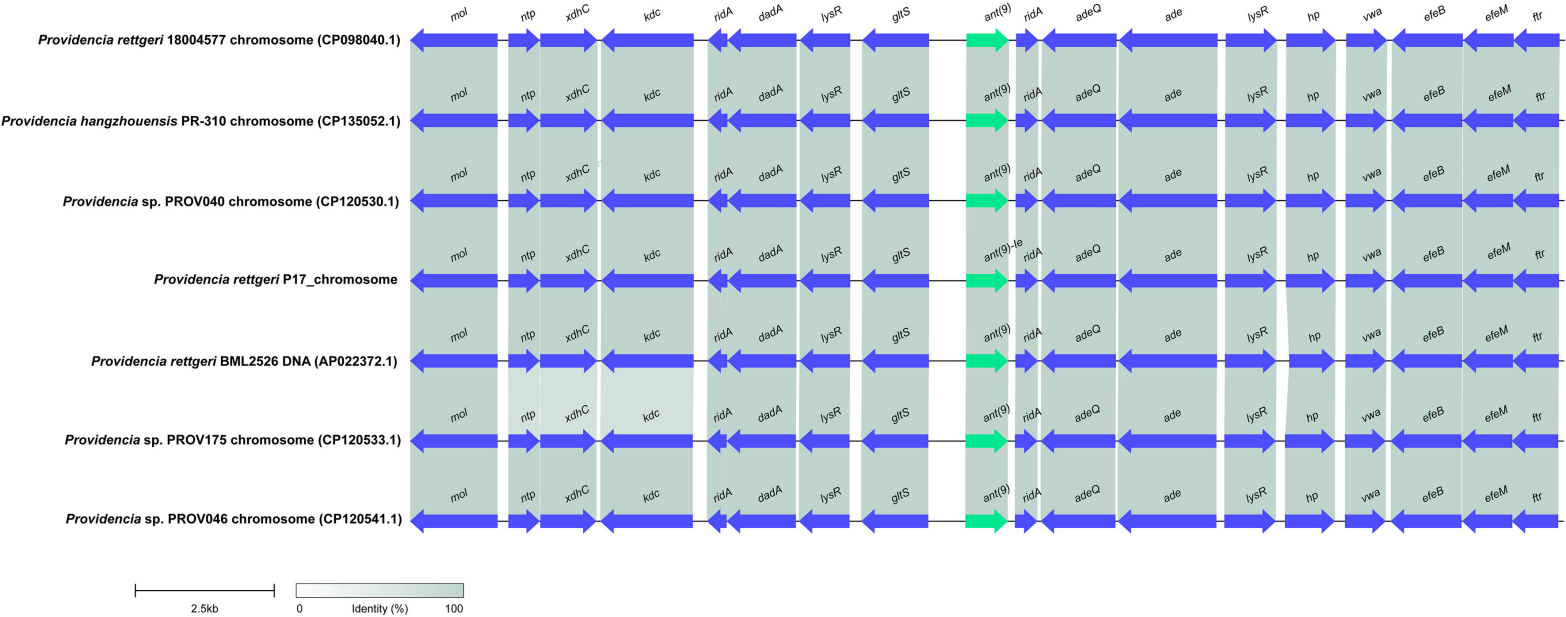
Genetic backgrounds of *ant(9)-Ie*(-like) genes. Genes encoding proteins with ≥ 80.0% aa identity are shaded in gray. The *ant(9)-Ie*(-like) genes are shown in green.

*ant(9)-Ie* is located on the chromosome of *P. rettgeri* P17. We retrieved 42 sequences (including the sequence from this work) of approximately 20 kb in length, each centered on an *ant(9)-Ie* homolog, from the NCBI nucleotide database to investigate the genetic backgrounds of *ant(9)-Ie* and its homologs. A comparative genomic analysis revealed that these 20-kb sequences presented nucleotide similarities ranging from 100.0% to 95.15% with the sequence of this work (**Supplementary Table 4**). Multiple sequence alignment of seven 20-kb sequences, including the sequence from this study, showed a high degree of similarity in both their gene composition and gene order. These observations collectively indicate the strong conservation of the genomic regions encoding *ant(9)-Ie-*like genes across members of the genus *Providencia*. Sequencing analysis revealed several functional categories associated with the gene flanking regions, including genes encoding transcriptional regulators (*Lys*R family), purine metabolism-related proteins (*xdh*C and *ade*Q), amino acid metabolism-related proteins (*rid*A) and iron homeostasis-related factors (*efe*M, *efe*B, and *ftr*). Additionally, screening of this region confirmed the absence of mobile genetic elements (**Figure 1**).

### Phylogenetic relationship and comparison of the structure of ANT(9)-Ie with other functionally characterized proteins

**Figure 2:**
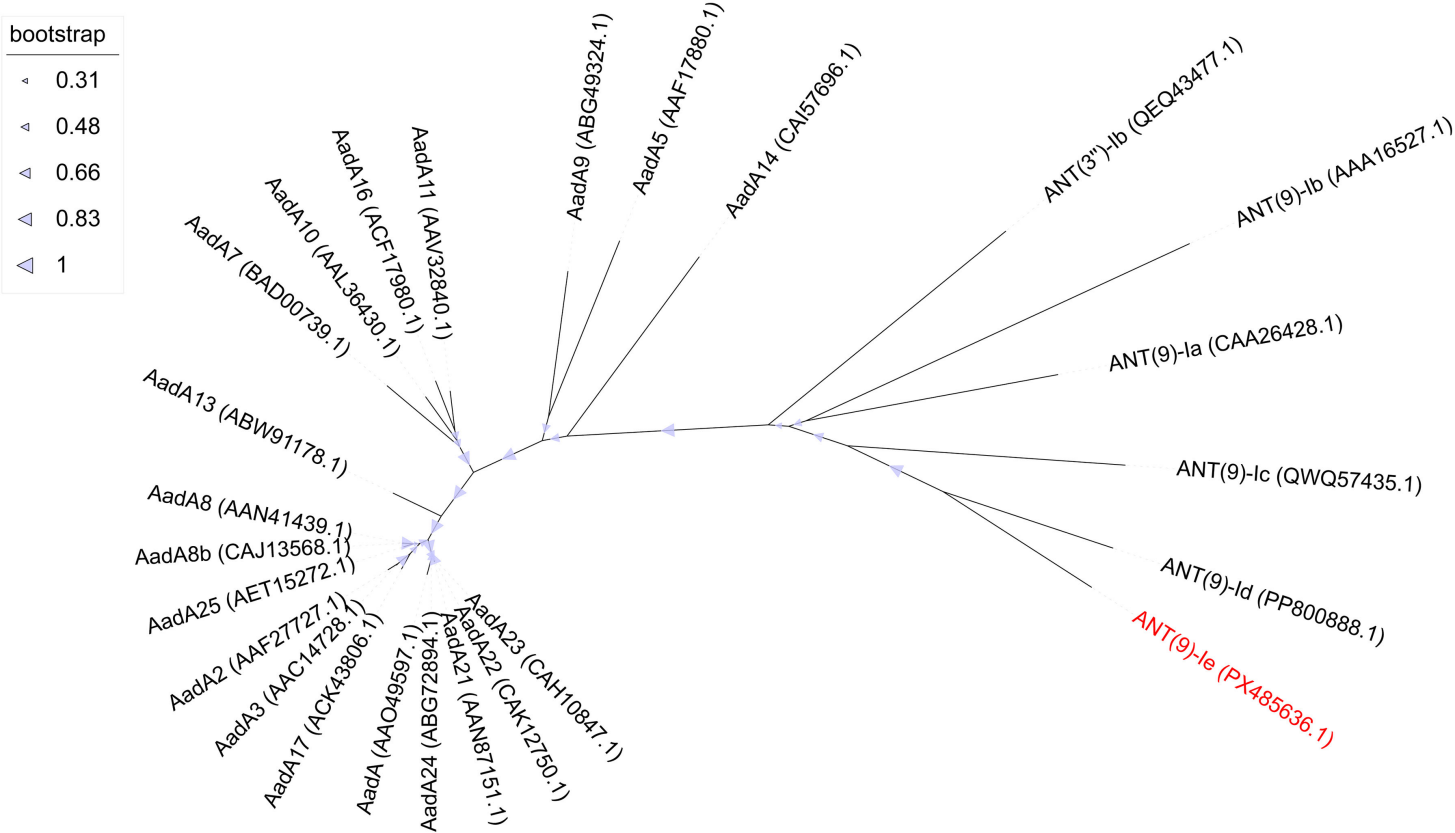
Phylogenetic tree illustrating the association between ANT(9)-Ie and other functionally characterized ANTs. The number of bootstrap replicates was 1000. ANT is the abbreviation for the aminoglycoside nucleotidyltransferase, and the accession numbers of the proteins are provided in parentheses.

A total of 24 functionally characterized proteins from the CARD database showing ≥ 36.0% aa sequence similarity to ANT(9)-Ie were retrieved to elucidate the evolutionary relationship of ANT(9)-Ie with resistance proteins. Among them, ANT(9)-Ie exhibited the highest aa similarity (58.67%) with ANT(9)-Id (PP800888.1) from an unclassified strain (*Providencia* sp. TYF-12) of the same genus as that studied in this work (*Providencia*). The other two proteins with > 45.0% aa similarity were ANT(9)-Ic (QWQ57435.1, 49.33%) from *Brucella intermedia* and ANT(9)-Ia (CAA26428.1, 45.49%) from *Staphylococcus aureus*. The evolutionary tree revealed that ANT(9)-Ie clustered close to the ANT(9) proteins (**Figure 2**). Collectively, these results strongly support the classification of the novel resistance protein as a member of the ANT(9)-I family.

**Figure 3:**
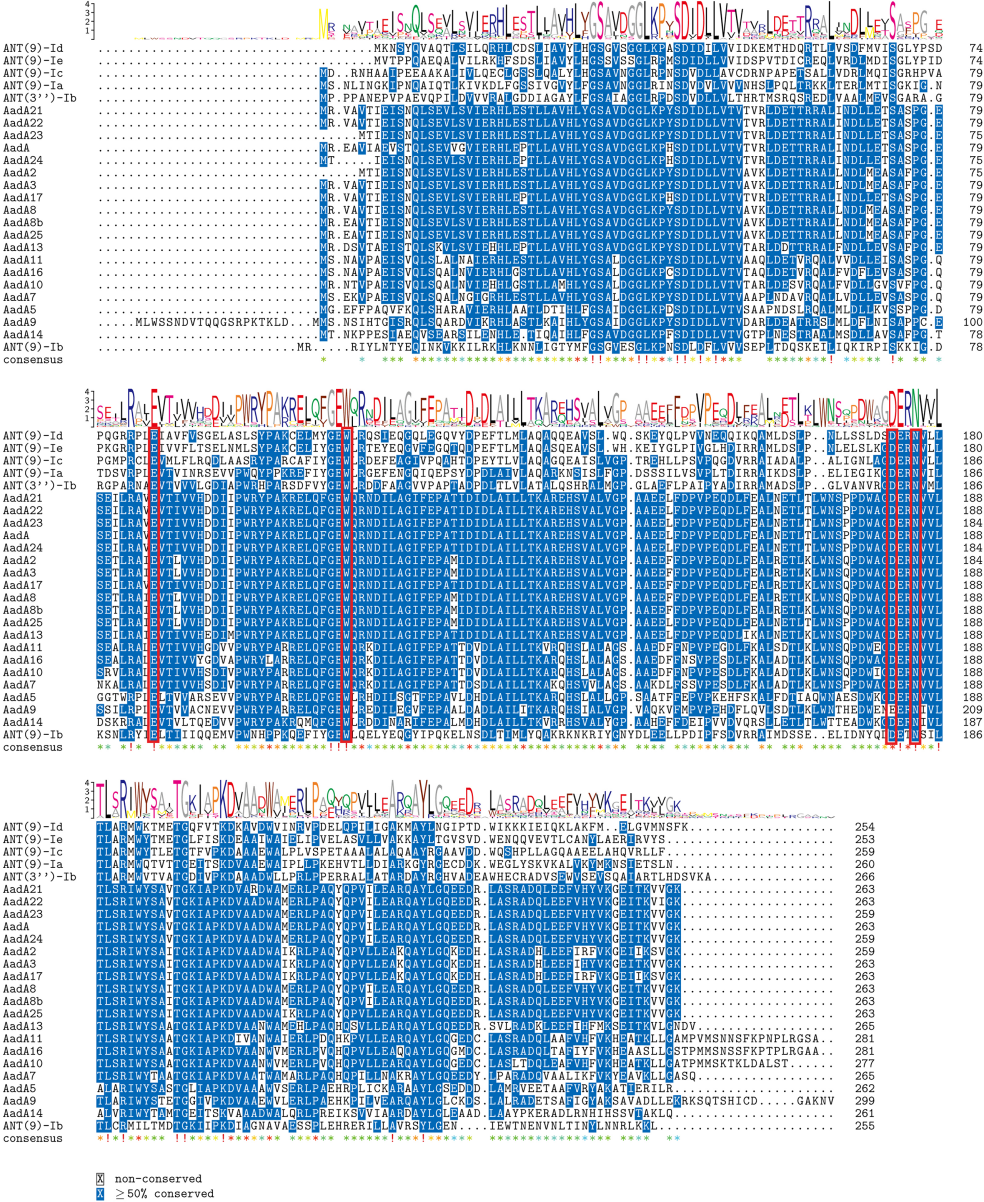
Comparisons of the primary and secondary structures of ANT(9)-Ie and its homologs. The blue frames indicate functional residues. The numbers on the left denote the corresponding sequence length. The accession numbers of the proteins are the same as those in **Figure 2**.

We analyzed the structural characteristics of the protein ANT(9)-Ie by comparing the primary and secondary structures of ANT(9)-Ie with those of its close homologs. The results indicated that the secondary structure and functionally essential residues of the ANT(9) protein were conserved in ANT(9)-Ie. For AadA, the residues E87, W112, D182, and H185/N are critical determinants of its ability to adenylate spectinomycin (Stern et al., 2018). In ANT(9)-Ie, these residues remained unchanged (E82, W107, D172 and N175) (**Figure 3**).

According to the 3D structural analysis, it revealed that Ant(9)-Ie had a narrower pocket and a shorter binding distance (4.1 Å) with spectinomycin (**Supplementary Figure 2A**), indicating a lower catalytic constant (*k*_cat_) and a stronger binding power with a lower *K*_m_ value. In contrast, Ant(9)-Ic (**Supplementary Figure 2B**) had a wider and more flexible catalytic pocket, indicating a higher catalytic constant (*k*_cat_), and had a longer binding distance (6.3 Å) between ATP and the substrate spectinomycin, showing a weaker binding power with a higher *K*_m_ value. As for the Ant(9)-Id (**Supplementary Figure 2C**), it showed a similar binding distance (6.3 Å) between ATP and the substrate spectinomycin with that of Ant(9)-Ic and had a similar narrow pocket with that of Ant(9)-Ie (**Table 4**, **Supplementary Table 5**).

**Table 4:** Kinetic parameters of ANT(9)-Ie, ANT(9)-Ib, ANT(9)-Ic and ANT(9)-Id.

Enzyme	Substrate	*k*_cat_ (s^-1^)	*K*_m_ (μM)	*k*_cat_/*K*_m_ (M^-1^·s^-1^)
ANT(9)-Ie	Spectinomycin	(8.82 ± 1.42) × 10^-3^	11.10 ± 3.62	(8.22 ± 1.24) × 10^2^
(This work)	Streptomycin	NH*	NH*	NH*
	Amikacin	NH*	NH*	NH*
	Kanamycin	NH*	NH*	NH*
	Gentamicin	NH*	NH*	NH*
ANT(9)-Ib (LeBlanc et al., 1991)	Spectinomycin	2.6 ± 0.2	33.56 ± 8.1	(8.3 ± 2.1) × 10^4^
ANT(9)-Ic (Sheng et al., 2023)	Spectinomycin	1.2 ± 0.4	44.83 ± 6.2	(2.8 ± 0.6) × 10^4^
ANT(9)-Id (Yu et al., 2024)	Spectinomycin	231.57 ± 59.80	8.94 ± 2.50	(2.62 ± 0.3) × 10^7^

*NH, no detectable hydrolysis.

### Kinetic characteristics of ANT(9)-Ie

The ORF of *ant(9)-Ie* was cloned to further study the characteristics of its enzymatic activity (**Supplementary Figure 1B**). The ANT(9)-Ie protein was overexpressed (**Supplementary Figure 3A**) and purified (**Supplementary Figure 3B**). According to the MIC results, the recombinant cells harboring *ant(9)-Ie* were resistant only to spectinomycin. The results of the enzyme kinetic analysis were consistent with the MIC results. Mirroring the resistance phenotype, ANT(9)-Ie was able to specifically adenylate spectinomycin, with a *K*_m_ of 11.10 ± 3.62 μM and a *k*_cat_/*K*_m_ of (8.22 ± 1.24) × 10^2^ M^-1^·s^-1^ (**Supplementary Figure 4**). However, the adenylation of streptomycin, amikacin, kanamycin, or gentamicin was not observed (**Table 4**). The *K*_m_ value of Ant(9)-Ie was similar to that of Ant(9)-Id (11.10 ± 3.62 vs. 8.94 ± 2.50 μM) (Yu et al., 2024) but lower than that of Ant(9)-Ib/Ant(9)-Ic (11.10 ± 3.62 vs. 33.56 ± 8.1/44.83 ± 6.2 μM) (LeBlanc et al., 1991; Kanchugal P and Selmer, 2020; Sheng et al., 2023). Furthermore, Ant(9)-Ie had a much lower adenylation efficiency for spectinomycin than Ant(9)-Ib/Ant(9)-Ic/Ant(9)-Id did [*k*_cat_/*K*_m_, (8.22 ± 1.24) × 10^2^ vs. (8.3 ± 2.1) × 10^4^/(2.8 ± 0.6) × 10^4^/(2.62 ± 0.3) × 10^7^ M^-1^·s^-1^] (LeBlanc et al., 1991; Kanchugal P and Selmer, 2020; Sheng et al., 2023; Yu et al., 2024).

## Conclusions

In the current study, we report the discovery and functional characterization of a novel aminoglycoside resistance gene, *ant(9)-Ie*, located on the chromosome of a *Providencia rettgeri* strain isolated from the environment that confers resistance to spectinomycin. The *ant(9)-Ie* gene and its close relatives were distributed among bacteria of the genus *Providencia* isolated from different sources, including human clinical and animal specimens. These findings indicate that the discovery of new resistance mechanisms in environmental microbes may contribute to a better understanding of resistance dissemination and its clinical implications.

## Data Availability

The datasets presented in this study can be found in online repositories. The names of the repository/repositories and accession number(s) can be found in the article/**Supplementary Material**.

## Supplementary material

Supplementary Figure 1 Orientation of the cloned genes.

Supplementary Figure 2 Ant(9)-I enzyme–antibiotic docking analysis.

Supplementary Figure 3 Protein expression and purification.

Supplementary Figure 4 Kinetic parameters and Michaelis-Menten plots of ANT(9)-Ie.

Supplementary Table 1 The primers used in this study.

Supplementary Table 2 Whole-genome sequencing and genome assembly quality metrics of *Providencia rettgeri* P17.

Supplementary Table 3 Sources of the *ant(9)-Ie*(-like) genes.

Supplementary Table 4 Similarity of the *ant(9)-Ie*(-like) gene encoding regions.

Supplementary Table 5 Ant(9)-I enzyme–antibiotic docking analysis.

## References

[B1] Aradi K. Di Giorgio A. Duca M. (2020). Corrigendum: aminoglycoside conjugation for RNA targeting: antimicrobials and beyond. Chem. Eur. J. 26, 17275–17282. doi: 10.1002/chem.202004916. PMID: 33333619

[B2] Auch A. F. Klenk H.-P. Göker M. (2010). Standard operating procedure for calculating genome-to-genome distances based on high-scoring segment pairs. Stand. Genomic Sci. 2, 142–148. doi: 10.4056/sigs.541628. PMID: 21304686 PMC3035261

[B3] Chen Q. Zhou W. Qian C. Shen K. Zhu X. Zhou D. . (2019). OXA-830, a novel chromosomally encoded extended-spectrum class D β-lactamase in Aeromonas simiae. Front. Microbiol. 10, 2732. doi: 10.3389/fmicb.2019.02732. PMID: 31849884 PMC6902050

[B4] Ciufo S. Kannan S. Sharma S. Badretdin A. Clark K. Turner S. . (2018). Using average nucleotide identity to improve taxonomic assignments in prokaryotic genomes at the NCBI. Int. J. Syst. Evol. Microbiol. 68, 2386–2392. doi: 10.1099/ijsem.0.002809. PMID: 29792589 PMC6978984

[B5] CLSI (2024). Methods for dilution antimicrobial susceptibility tests for bacteria that grow aerobically CLSI standard M07 (Wayne, PA: Clinical and Laboratory Standards Institute).

[B6] CLSI (2025). Performance standards for antimicrobial susceptibility testing. 36th ed. CLSI standard M100. (Wayne, PA: Clinical and Laboratory Standards Institute).

[B7] Eberhardt J. Santos-Martins D. Tillack A. F. Forli S. (2021). AutoDock Vina 1.2.0: new docking methods, expanded force field, and Python bindings. J. Chem. Inf. Model. 61, 3891–3898. doi: 10.1021/acs.jcim.1c00203. PMID: 34278794 PMC10683950

[B8] Gilchrist C. L. M. Chooi Y.-H. (2021). clinker & clustermap.js: automatic generation of gene cluster comparison figures. Bioinformatics 37, 2473–2475. doi: 10.1093/bioinformatics/btab007. PMID: 33459763

[B9] Harris P. N. A. Ferguson J. K. (2012). Antibiotic therapy for inducible AmpC β-lactamase-producing Gram-negative bacilli: what are the alternatives to carbapenems, quinolones and aminoglycosides? Int. J. Antimicrob. Agents 40, 297–305. doi: 10.1016/j.ijantimicag.2012.06.004. PMID: 22824371

[B10] ISO (2019). Susceptibility testing of infectious agents and evaluation of performance of antimicrobial susceptibility test devices—Part 1: broth micro-dilution reference method for testing the *in vitro* activity of antimicrobial agents against rapidly growing aerobic bacteria involved in infectious diseases ISO 20776-1 (Geneva: International Organization for Standardization).

[B11] Jain C. Rodriguez-R L. M. Phillippy A. M. Konstantinidis K. T. Aluru S. (2018). High throughput ANI analysis of 90K prokaryotic genomes reveals clear species boundaries. Nat. Commun. 9, 5114. doi: 10.1038/s41467-018-07641-9. PMID: 30504855 PMC6269478

[B12] Janson G. Paiardini A. (2021). PyMod 3: a complete suite for structural bioinformatics in PyMOL. Bioinformatics 37, 1471–1472. doi: 10.1093/bioinformatics/btaa849. PMID: 33010156

[B13] Jumper J. Evans R. Pritzel A. Green T. Figurnov M. Ronneberger O. . (2021). Highly accurate protein structure prediction with AlphaFold. Nature 596, 583–589. doi: 10.1038/s41586-021-03819-2. PMID: 34265844 PMC8371605

[B14] Kanchugal P S. Selmer M. (2020). Structural recognition of spectinomycin by resistance enzyme ANT(9) from Enterococcus faecalis. Antimicrob. Agents Chemother. 64, e00371-20. doi: 10.1128/AAC.00371-20. PMID: 32253216 PMC7269491

[B15] Katoh K. Standley D. M. (2013). MAFFT multiple sequence alignment software version 7: improvements in performance and usability. Mol. Biol. Evol. 30, 772–780. doi: 10.1093/molbev/mst010. PMID: 23329690 PMC3603318

[B16] Konstantinidis K. T. Tiedje J. M. (2005). Genomic insights that advance the species definition for prokaryotes. Proc. Natl. Acad. Sci. 102, 2567–2572. doi: 10.1073/pnas.0409727102. PMID: 15701695 PMC549018

[B17] Kumar S. Stecher G. Li M. Knyaz C. Tamura K. (2018). MEGA X: molecular evolutionary genetics analysis across computing platforms. Mol. Biol. Evol. 35, 1547–1549. doi: 10.1093/molbev/msy096. PMID: 29722887 PMC5967553

[B18] Labby K. J. Garneau-Tsodikova S. (2013). Strategies to overcome the action of aminoglycoside-modifying enzymes for treating resistant bacterial infections. Future Med. Chem. 5, 1285–1309. doi: 10.4155/fmc.13.80. PMID: 23859208 PMC3819198

[B19] LeBlanc D. J. Lee L. N. Inamine J. M. (1991). Cloning and nucleotide base sequence analysis of a spectinomycin adenyltransferase AAD(9) determinant from Enterococcus faecalis. Antimicrob. Agents Chemother. 35, 1804–1810. doi: 10.1128/AAC.35.9.1804. PMID: 1659306 PMC245272

[B20] Linkevicius M. Witteveen S. Buzea M. Flonta M. Indreas M. Nica M. . (2024). Genomic surveillance detects interregional spread of New Delhi metallo-beta-lactamase-1-producing Providencia stuartii in hospitals, Romania, December 2021 to September 2023. Eurosurveillance 29, 1–10. doi: 10.2807/1560-7917.ES.2024.29.47.2400587. PMID: 39574389 PMC11583310

[B21] Liu M. Yi N. Wang X. Wang R. (2023). Analysis of resistance genes of carbapenem-resistant Providencia rettgeri using whole genome sequencing. BMC Microbiol. 23, 283. doi: 10.1186/s12866-023-03032-3. PMID: 37789331 PMC10546784

[B22] Magnet S. Blanchard J. S. (2005). Molecular insights into aminoglycoside action and resistance. Chem. Rev. 105, 477–498. doi: 10.1021/cr0301088. PMID: 15700953

[B23] Magnet S. Courvalin P. Lambert T. (2001). Resistance-nodulation-cell division-type efflux pump involved in aminoglycoside resistance in Acinetobacter baumannii strain BM4454. Antimicrob. Agents Chemother. 45, 3375–3380. doi: 10.1128/aac.45.12.3375-3380.2001. PMID: 11709311 PMC90840

[B24] Magnet S. Smith T.-A. Zheng R. Nordmann P. Blanchard J. S. (2003). Aminoglycoside resistance resulting from tight drug binding to an altered aminoglycoside acetyltransferase. Antimicrob. Agents Chemother. 47, 1577–1583. doi: 10.1128/aac.47.5.1577-1583.2003. PMID: 12709325 PMC153337

[B25] Malviya M. Kale-Pradhan P. Coyle M. Giuliano C. Johnson L. B. (2024). Clinical and drug resistance characteristics of Providencia infections. Microorganisms 12, 2085. doi: 10.3390/microorganisms12102085. PMID: 39458394 PMC11510300

[B26] Mirdita M. Schütze K. Moriwaki Y. Heo L. Ovchinnikov S. Steinegger M. (2022). ColabFold: making protein folding accessible to all. Nat. Methods 19, 679–682. doi: 10.1038/s41592-022-01488-1. PMID: 35637307 PMC9184281

[B27] Morris G. M. Huey R. Lindstrom W. Sanner M. F. Belew R. K. Goodsell D. S. (2009). AutoDock4 and AutoDockTools4: Automated docking with selective receptor flexibility. J. Comput. Chem. 30, 2785–2791. doi: 10.1002/jcc.21256 19399780 PMC2760638

[B28] Musser J. M. (1995). Antimicrobial agent resistance in Mycobacteria: molecular genetic insights. Clin. Microbiol. Rev. 8, 496–514. doi: 10.1128/cmr.8.4.496. PMID: 8665467 PMC172873

[B29] Nikaido H. (2003). Molecular basis of bacterial outer membrane permeability revisited. Microbiol. Mol. Biol. Rev. 67, 593–656. doi: 10.1128/mmbr.67.4.593-656.2003. PMID: 14665678 PMC309051

[B30] O’Hara C. M. Brenner F. W. Miller J. M. (2000). Classification, identification, and clinical significance of Proteus, Providencia, and Morganella. Clin. Microbiol. Rev. 13, 534–546. doi: 10.1128/cmr.13.4.534. PMID: 11023955 PMC88947

[B31] Richter M. Rosselló-Móra R. (2009). Shifting the genomic gold standard for the prokaryotic species definition. Proc. Natl. Acad. Sci. 106, 19126–19131. doi: 10.1073/pnas.0906412106. PMID: 19855009 PMC2776425

[B32] Shah M. Kathiiko C. Wada A. Odoyo E. Bundi M. Miringu G. . (2016). Prevalence, seasonal variation, and antibiotic resistance pattern of enteric bacterial pathogens among hospitalized diarrheic children in suburban regions of central Kenya. Trop. Med. Health 44, 39–46. doi: 10.1186/s41182-016-0038-1. PMID: 27942243 PMC5126808

[B33] Shakil S. Khan R. Zarrilli R. Khan A. U. (2008). Aminoglycosides versus bacteria – a description of the action, resistance mechanism, and nosocomial battleground. J. Biomed. Sci. 15, 5–14. doi: 10.1007/s11373-007-9194-y. PMID: 17657587

[B34] Sharma R. Rajni E. Garg V. K. Vohra R. Jain S. S. (2022). Providencia causing urinary tract infections: are we reaching a dead end? Indian J. Crit. Care Med. 26, 448–453. doi: 10.5005/jp-journals-10071-24163. PMID: 35656046 PMC9067475

[B35] Sheng X. Lu W. Li A. Lu J. Song C. Xu J. . (2023). ANT(9)-Ic, a novel chromosomally encoded aminoglycoside nucleotidyltransferase from Brucella intermedia. Microbiol. Spectr. 11, e00620-23. doi: 10.1128/spectrum.00620-23. PMID: 37039640 PMC10269693

[B36] Stern A. L. Van Der Verren S. E. Kanchugal P S. Näsvall J. Gutiérrez-de-Terán H. Selmer M. (2018). Structural mechanism of AadA, a dual-specificity aminoglycoside adenylyltransferase from Salmonella enterica. J. Biol. Chem. 293, 11481–11490. doi: 10.1074/jbc.ra118.003989. PMID: 29871922 PMC6065190

[B37] Stothard P. (2000). The sequence manipulation suite: JavaScript programs for analyzing and formatting protein and DNA sequences. BioTechniques 28, 1102–1104. doi: 10.2144/00286ir01. PMID: 10868275

[B38] Valiatti T. B. Santos F. F. Bessa-Neto F. O. Veiga R. Simionatto S. De Almeida Souza G. H. . (2024). Emergence of multidrug-resistant Providencia rettgeri clone in food-producing animals: a public health threat. One Health 19, 100887. doi: 10.1016/j.onehlt.2024.100887. PMID: 39323428 PMC11422129

[B39] Wendlandt S. Li B. Lozano C. Ma Z. Torres C. Schwarz S. (2013). Identification of the novel spectinomycin resistance gene spw in methicillin-resistant and methicillin-susceptible Staphylococcus aureus of human and animal origin. J. Antimicrob. Chemother. 68, 1679–1680. doi: 10.1093/jac/dkt081. PMID: 23511231

[B40] Yarlagadda V. Medina R. Wright G. D. (2020). Venturicidin A, a membrane-active natural product inhibitor of ATP synthase potentiates aminoglycoside antibiotics. Sci. Rep. 10, 8134. doi: 10.1038/s41598-020-64756-0. PMID: 32424122 PMC7235042

[B41] Yu Y. Zhang R. Pan W. Sheng X. Chen S. Wang J. . (2024). Identification and characterization of a novel chromosome-encoded aminoglycoside O-nucleotidyltransferase gene, ant(9)-Id, in Providencia sp. TYF-12 isolated from the marine fish intestine. Front. Microbiol. 15, 1475172. doi: 10.3389/fmicb.2024.1475172. PMID: 39726966 PMC11669914

