# Supplementary figures and images for: Discovery and characterization of a novel chromosomally encoded aminoglycoside O-nucleotidyltransferase gene, designated ant(9)-Ie, in a strain of Providencia
Source: Front Cell Infect Microbiol. 2026 Jun 2;16:1772530. doi: 10.3389/fcimb.2026.1772530 (PMC13268890; doi:10.3389/fcimb.2026.1772530)

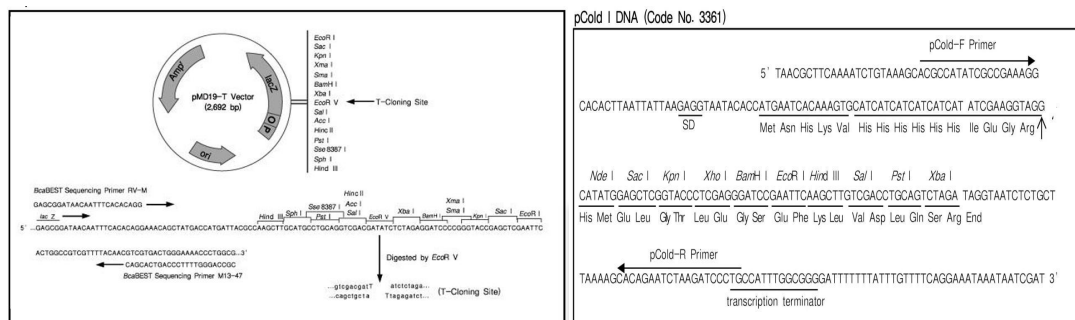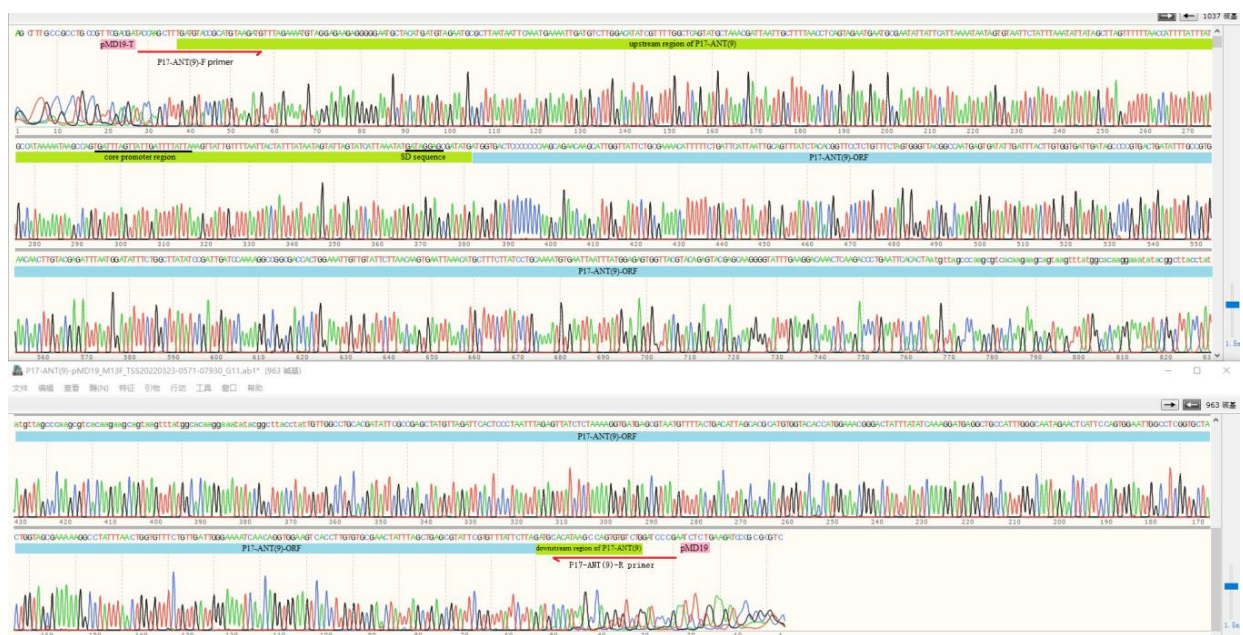

Supplement: Supplementary Figure 1 — Orientation of the cloned genes. [file DataSheet1.pdf]
