# Supplementary material for: Discovery and characterization of a novel chromosomally encoded aminoglycoside O-nucleotidyltransferase gene, designated ant(9)-Ie, in a strain of Providencia
Source: Front Cell Infect Microbiol. 2026 Jun 2;16:1772530. doi: 10.3389/fcimb.2026.1772530 (PMC13268890; doi:10.3389/fcimb.2026.1772530)
Supplement: Supplementary Figure 2 — Ant(9)-I enzyme–antibiotic docking analysis. [file DataSheet2.pdf]

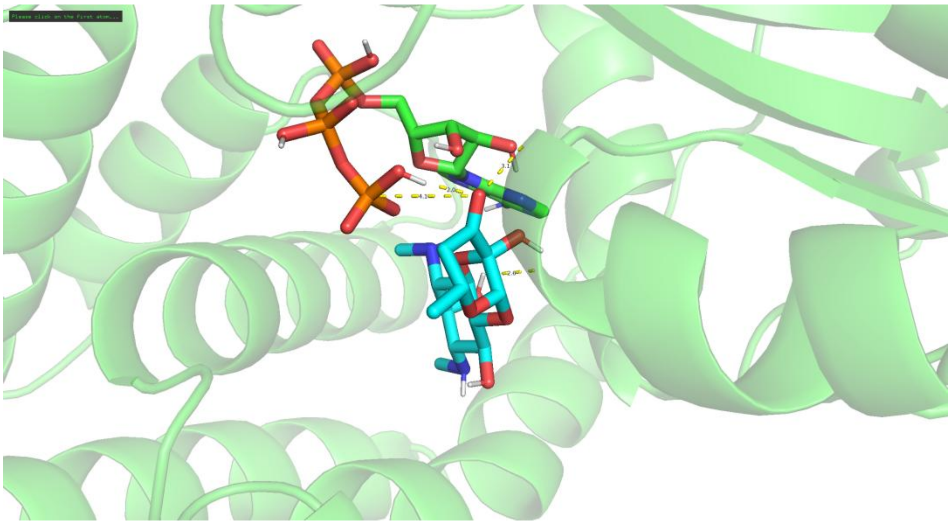

Figure 2A. ANT9_Ie_Distance_4.1_NoShadow_Mg

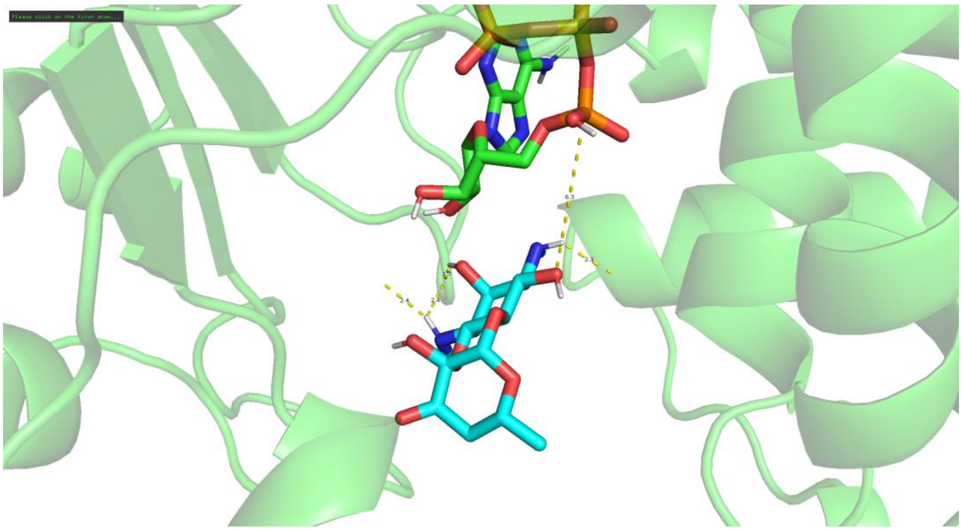

Figure 2B. ANT9_Ic_Distance_6.3_NoShadow_Mg

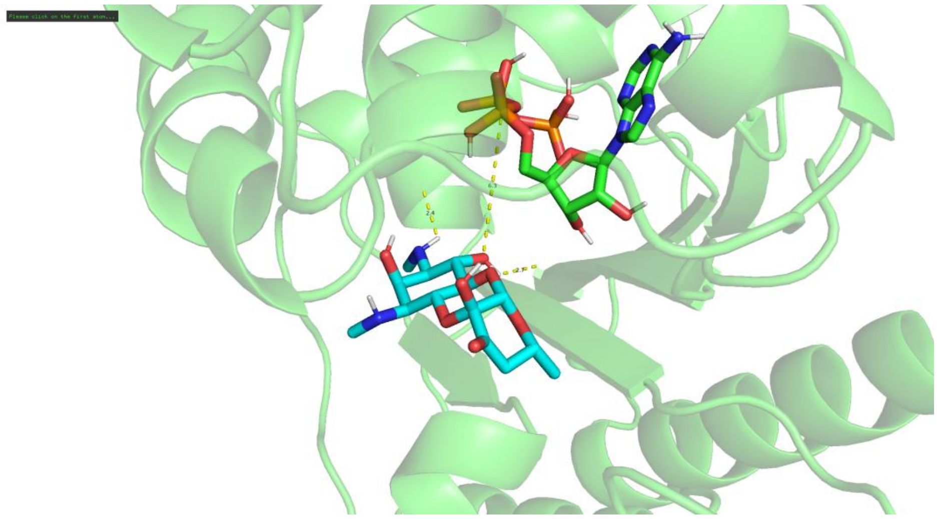

Figure 2C. ANT9_Id_Distance_6.3_NoShadow_Mg
