# Supplementary material for: Discovery and characterization of a novel chromosomally encoded aminoglycoside O-nucleotidyltransferase gene, designated ant(9)-Ie, in a strain of Providencia
Source: Front Cell Infect Microbiol. 2026 Jun 2;16:1772530. doi: 10.3389/fcimb.2026.1772530 (PMC13268890; doi:10.3389/fcimb.2026.1772530)
Supplement: Supplementary Figure 3 — Protein expression and purification. [file DataSheet3.pdf]

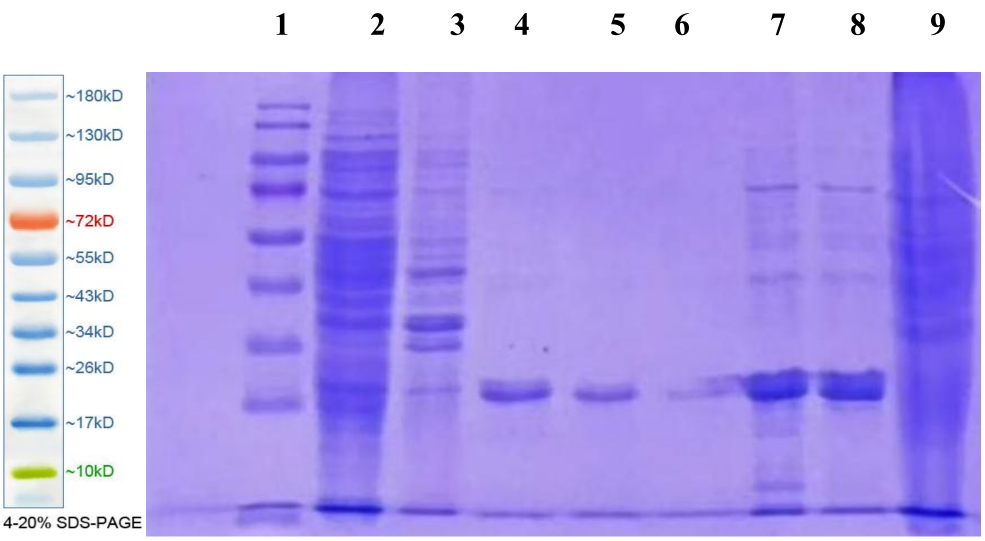

Figure S2A Results of SDS-PAGE electrophoresis of proteins eluted with imidazole salt buffer at different concentrations. 1: PageRuler™ pre-stained protein marker (10–180 kDa); 2: NTA eluent with 0 mM imidazole; 3: NTA elution with 50 mM imidazole; 4: Eluent with 100 mM imidazole; 5: Eluent with 250 mM imidazole; 6: Eluent with 500 mM imidazole; 7–8: Supernatant of lysed BL21/pCold Ⅰ-ant(9)-P17 strain after expression; 9: Precipitate of lysed BL21/pCold Ⅰ-ant(9)-P17 strain after expression.

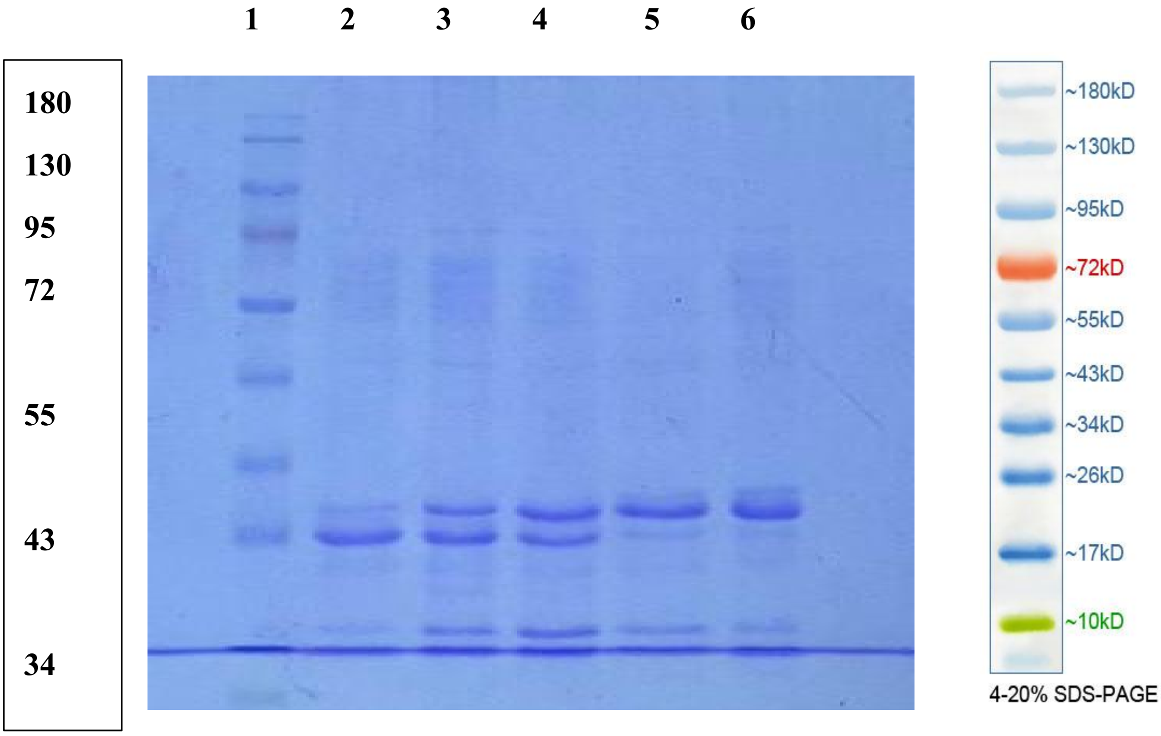

Figure S2A SDS-PAGE analysis of purified proteins after thrombin degestion. 1, PageRuler™ Prestained Protein Ladder (10–180 kDa); 2, Digested for 2.0 h; 3, Digested for 1.5 h; 4, Digested for 1.0 h; 5, Digested for 0.5 h; 6, Undigested control.
