# Supplementary material for: Discovery and characterization of a novel chromosomally encoded aminoglycoside O-nucleotidyltransferase gene, designated ant(9)-Ie, in a strain of Providencia
Source: Front Cell Infect Microbiol. 2026 Jun 2;16:1772530. doi: 10.3389/fcimb.2026.1772530 (PMC13268890; doi:10.3389/fcimb.2026.1772530)

Kinetic parameters and Michaelis-Menten plots of ANT(9)-Ie.

	average	1	2	3
kcat				
Best-fit values				
Et (μM)	0.075	0.075	0.075	0.075
k_{cat}	0.008822333	0.01041	0.008385	0.007672
K_m (μM)	11.097	15.17	9.876	8.245
V_{max}		0.0007805	0.0006289	0.0005754
95% CI (profile likelihood)				
k_{cat}		0.007766 - 0.01567	0.006830 - 0.01079	0.005719 - 0.01152
K_m		7.591 - 31.25	5.429 - 17.17	2.697 - 20.61
Goodness of Fit				
Degrees of Freedom		4	4	3
R squared		0.9523	0.9523	0.9194
Sum of Squares		1.917E-09	1.169E-09	1.603E-09
Sy.x		0.00002189	0.0000171	0.00002311
Constraints				
Et		Et = 0.075	Et = 0.075	Et = 0.075
Number of points				
# of X values		6	6	6
# Y values analyzed		6	6	5
k_{cat}/K_m (M-1·s-1)	821.91803	686.2228082	849.0279465	930.5033354

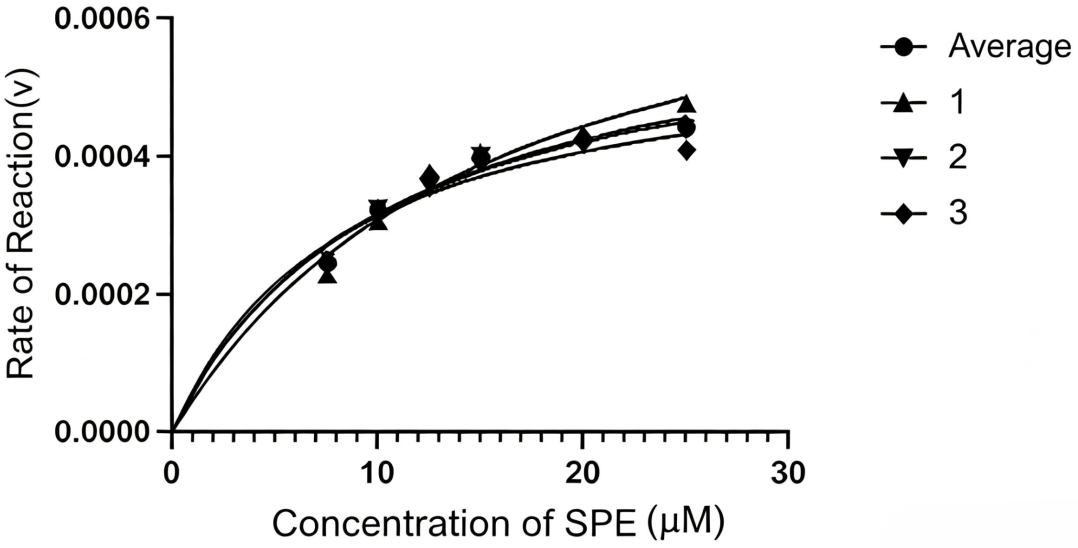

Supplement: Supplementary Figure 4 — Kinetic parameters and Michaelis-Menten plots of ANT(9)-Ie. [file DataSheet4.pdf]
