# Supplementary material for: Discovery and characterization of a novel chromosomally encoded aminoglycoside O-nucleotidyltransferase gene, designated ant(9)-Ie, in a strain of Providencia
Source: Front Cell Infect Microbiol. 2026 Jun 2;16:1772530. doi: 10.3389/fcimb.2026.1772530 (PMC13268890; doi:10.3389/fcimb.2026.1772530)
Supplement: Supplementary Table 1 — The primers used in this study. [file Table1.docx]

## Table S1. Primers used to clone the *ant(9)-Ie* gene.

| Primer^1^ | Sequence (5'→3') | Restriction endonuclease | Vector | Annealing temperature (℃) | Amplicon size (bp) |
| --- | --- | --- | --- | --- | --- |
| pro-*ant(9)-Ie*-F | CCAAGCTTTGATGTACCGCATGTAAGATG | *Hind*Ⅲ | pMD19 | 54 | 1,145 |
| pro-ant(9)*-Ie*-R | CGGGATCCAGACACACTGGCTTATGTG | *Bam*HI | pMD19 |  | 1,145 |
| orf-*ant(9)-Ie*-F | GGATCCCTGGTGCCGCGCGGCAGCATGGTGACTCCCCCCCAA | *Bam*HI *+* Thrombin | pCold I | 60 | 792 |
| orf-ant(9)*-Ie*-R | TCTAGACTAAGAATAAACACGAATACGCTCAGCTAAATA | *Xba*I | pCold I |  | 792 |

^1^ Primers started with “pro” were used to clone the *ant(9)*gene with its promoter region; primers started with “orf” were used to clone the ORF of the *ant(9)* gene.
